# ﻿Insights into phylogenetic relationships and gene rearrangements: complete mitogenomes of two sympatric species in the genus *Rana* (Anura, Ranidae)

**DOI:** 10.3897/zookeys.1216.131847

**Published:** 2024-10-21

**Authors:** Jingfang Li, Mei Xie, Fangpeng Zhang, Juan Shu, Jun Zhang, Zhinuo Zhang, Hongmei Xiang, Wansheng Jiang

**Affiliations:** 1 College of Biology and Environmental Sciences, Jishou University, Jishou 416000, China Jishou University Jishou China; 2 National and Local United Engineering Laboratory of Integrative Utilization Technology of Eucommia ulmoides, Jishou University, Zhangjiajie 427000, China Jishou University Zhangjiajie China; 3 Zhangjiajie National Forest Park, Zhangjiajie 427400, China Zhangjiajie National Forest Park Zhangjiajie China

**Keywords:** China, genome, mitochondrial, phylogeny, Ranine, Zhangjiajie

## Abstract

Mitochondrial genomes (also known as mitogenomes) serve as valuable molecular markers and have found widespread applications in molecular biology and ecology. There is abundant sequence variation in vertebrate mitogenomes, and occasionally, they exhibit gene rearrangements. In this study, two Chinese endemic *Rana* species, *Ranajiemuxiensis* and *Ranahanluica*, were sequenced and analyzed to obtain their complete mitogenomes. The two species were sympatrically distributed in the Zhangjiajie National Forest Park, in Wulingyuan District, Zhangjiajie City, Hunan Province, China. The mitogenome of *R.jiemuxiensis* was 17,506 bp, while that of *R.hanluica* was 17,505 bp, each comprising 13 protein-coding genes (PCGs), 22 transfer RNA genes (tRNAs), two ribosomal RNA genes (rRNAs), and a non-coding control region (D-loop). The gene content, nucleotide composition, and evolutionary rates of each mitogenome were analyzed and compared with those of congeners. A phylogenetic analysis based on 22 mitogenomes in *Rana* revealed that the two sympatric species were in two different lineages, indicating that they were genetically separated to a certain extent. Three types of gene rearrangement patterns were identified when examining the gene orders of the 22 *Rana* mitogenomes. Most of the species shared a second and dominant gene rearrangement pattern that originated from the first ancient pattern. A “tandem duplication – multiple deletion” hypothesis was proposed to explain the evolution of these different gene rearrangement patterns. This study provided valuable data references and enhanced our understanding of the phylogenetic implications and gene rearrangements of *Rana* species.

## ﻿Introduction

The amphibian genus *Rana* Linnaeus, 1758, commonly referred to as the “wood frog” or “brown frog,” represents an early-established group typified by the European wood frog, *Ranatemporaria* Linnaeus, 1758. Over the past two decades, there has been significant taxonomic restructuring within the genus *Rana* and its ranine counterparts. According to the Amphibian Species of the World online database, the currently reclassified *Rana* encompasses 52 species distributed throughout temperate Eurasia into Indochina (Frost 2024). Notably, China hosts the largest number of *Rana* species, totaling 28, which are extensively dispersed across almost every province of the country, ranging from the north in the Heilongjiang River basin, to the south in Hainan Island, and from the west in the Tibetan plateau, to the east in the East China coastal plain (AmphibiaChina 2024).

Numerous scientific investigations concerning *Rana* have focused on taxonomy and phylogeny, as well as the discovery of new species and insights into their ecological behaviors. The first discovery of a *Rana* species in China took place in the Qinling Mountains during the late 19^th^ century, officially named as *Ranachensinensis* David, 1875 (Boulenger 1920). However, for a long time, the Chinese wood frog *R.chensinensis* was often considered a synonym or subspecies of the European wood frog *R.temporaria* due to their morphological similarities (Liu and Hu 1961). The introduction of new phenotypic techniques and genetic tools, such as microstructure analysis, chromosome karyotyping, cytogenetics, protein analysis, isoenzymes, and DNA sequencing, has helped clarify many problematic species, identify new species, and gradually reveal phylogenetic relationships within wood frog groups (Wu 1981). For instance, Che et al. (2007) identified four well-supported clades of East Asian wood frogs through phylogenetic reconstructions using mitochondrial genes, showing significant geographic separations in the distribution patterns of some clades. Subsequently, Zhou et al. (2017) classified *Rana* species in East Asia into four species groups: *R.japonica* Boulenger, 1879, *R.maoershanensis* Lu, Li & Jiang, 2007, *R.chensinensis* and *R.amurensis* Boulenger, 1886. Their research emphasized that the *R.maoershanensis* and *R.japonica* species groups formed a monophyletic clade known as the Southern Chinese wood frogs, where the *R.japonica* species group could be further divided into *R.japonica*, *R.chaochiaoensis* Liu, 1946, and other species of the *R.longicrus* Stejneger, 1898 species group, each linked to distinct geographical regions.

*Ranahanluica* Shen, Jiang & Yang, 2007 and *Ranajiemuxiensis* Yan, Jiang, Chen, Fang, Jin, Li, Wang, Murphy, Che & Zhang, 2011 are two species belonging to the *R.longicrus* species group (Fei et al. 2009; Wan et al. 2020), which constitutes the largest species group within the Southern Chinese Wood Frogs. Recent studies focusing on the *R.longicrus* species group have revealed the presence of several new species in China, highlighting the incomplete understanding of this particular group of *Rana* (Pope and Boring 1940; Yan et al. 2011). Although initially discovered in a localized area in Hunan Province, distribution records of *R.jiemuxiensis* and *R.hanluica* exhibit different patterns. *Ranajiemuxiensis* has a relatively restricted distribution, being found only in the surrounding areas of its type locality, the Jiemuxi National Nature Reserve in Yuanling County (Yan et al. 2011). In contrast, the distribution range of *R.hanluica* extends beyond its type locality in Yangmingshan, Shuangpai County (Zhu et al. 2018), encompassing neighboring provinces such as Guizhou, Guangxi, Jiangxi, and even Zhejiang Province (AmphibiaChina 2024). Notably, *R.jiemuxiensis* and *R.hanluica* coexist in the Zhangjiajie National Forest Park, representing the northernmost distribution zones and likely the only coexisting area for both species to date. The reasons for the coexistence of these closely related species are multifaceted, likely stemming from their long-term evolutionary history. Factors contributing to their coexistence may include reproductive isolation, as well as potential genetic and niche differentiations. One factor facilitating their coexistence could be the non-synchronous breeding seasons. *Ranajiemuxiensis* typically breeds from late January to mid-March, with a peak breeding period in early March (Yan et al. 2011), whereas the breeding season of *R.hanluica* typically occurs in October, around the “hanlu” or “Cold Dew Festival” (Xia et al. 2022), one of the 24 traditional Chinese solar terms, which also inspired its species name “*hanluica*” (Shen et al. 2007).

The genetic differentiations between the two sympatric species, *R.jiemuxiensis* and *R.hanluica*, may play a crucial role in their coexistence, yet this aspect has received limited attention in previous studies. Furthermore, species within the Ranoidae family typically exhibit gene rearrangements in their mitogenomes (Igawa et al. 2008), which could further contribute to genetic differentiation and potentially influence their coexistence. In this study, we employed next-generation sequencing technology to sequence and characterize the complete mitochondrial genome of *R.jiemuxiensis* and *R.hanluica*. We conducted comparative analyses of these mitogenomes with those of closely related species, focusing on mitochondrial structures and features such as nucleotide composition, codon usage, and selection pressures. Additionally, we reconstructed the phylogenetic relationships using all available mitogenomes of the genus *Rana* obtained from NCBI and analyzed gene rearrangements within this group. The primary objective of this study is to provide novel insights into the phylogenetic implications and gene rearrangements of the two sympatric species, as well as other species within the genus *Rana*.

## ﻿Materials and methods

### ﻿Sample collection and sequencing

Samples of *R.jiemuxiensis* and *R.hanluica* were collected from Zhangjiajie National Forest Park (29°9′39″N, 110°24′58″E), located in the Wulingyuan District of Zhangjiajie City, Hunan Province, China. Permissions for the field survey were obtained for scientific purposes from the local administrations, and the sample collections and experimental protocols were approved by the Biomedical Ethics Committee of Jishou University (Approval No: JSDX–2024–0083). In accordance with the “3R principle” (Reduction, Replacement, and Refinement) as required by the National Ministry of Science and Technology (No. 398 [2006]), only one sample of each species was utilized. Specimens were euthanized humanely and preserved in 85% ethanol as voucher specimens. These specimens were deposited at the Molecular Ecology Laboratory, Zhangjiajie Campus, Jishou University (*R.jiemuxiensis*, voucher no. JWS20211037; *R.hanluica*, voucher no. JWS20211131). A small volume of liver tissue was used for molecular experiments. Total DNA was extracted using the DNeasy Blood & Tissue Kit (Qiagen, Hilden, Germany). DNA library construction was performed using the VAHTS Universal DNA Library Prep Kit for Illumina V3 (Vazyme, Nanjing, China). High-throughput sequencing was conducted on the DNBSEQ-T7 platform (Complete Genomics and MGI Tech, Shenzhen, China), generating approximately 30 Gb of raw reads with a read length of 150 bp for each sample.

### ﻿Mitogenome assembly, annotation, and character analyses

The complete mitogenomes of *R.jiemuxiensis* and *R.hanluica* were assembled using NOVOPlasty 4.3 (Dierckxsens et al. 2017), based on the raw reads generated through next-generation sequencing (NGS). The complete mitogenome and the CYTB gene (1140 bp) of *R.chensinensis* (NCBI accession No. KF898356) were used as the “reference” and “seed” sequences for assembling the mitogenomes of both *R.jiemuxiensis* and *R.hanluica*. The positions and orientations of protein-coding genes (PCGs), ribosomal RNA genes (rRNAs), transfer RNA genes (tRNAs), and the control region (D-loop) were annotated using the MITOS Web Server (http://mitos.bioinf.uni-leipzig.de/overview.py) (Bernt et al. 2013). The site and secondary structure of tRNAs were predicted using the tRNAscan-SE 1.21 online tool (http://lowelab.ucsc.edu/tRNAscan-SE/) (Chan and Lowe 2019). Visualization and circularization of the complete mitogenomes were performed using the CGview online server (https://cgview.ca/) (Grant and Stothard 2008).

The numbers of observed transitions (s) and transversions (v) for all the PCGs were plotted using DAMBE v7.3.11 (Xia 2018). Saturation was determined based on the values of *Iss* (simple index of substitution saturation) and *Iss. c* (critical *Iss* value). Other analyses, such as nucleotide composition, AT and GC skewing, codon usage frequency of PCGs, and determination of sequence genetic distances under the Kimura two-parameter (K2P) model, were conducted in MEGA 11.0. Relative synonymous codon usage (RSCU), nucleotide diversity (Pi), and the ratio of nonsynonymous substitution rate (Ka) to synonymous substitution rate (Ks) were calculated using DnaSP 6 (Rozas et al. 2017), with all stop codons removed before analysis.

### ﻿Phylogenetic analysis

The complete mitogenome sequences of the available species of the genus *Rana* were downloaded from NCBI. Twenty-two species in *Rana* were involved in the final dataset, including *R.jiemuxiensis* and *R.hanluica* that we sequenced. This dataset represented the most comprehensive set of mitogenome sequences available to date. An additional species, *Odorranajingdongensis* Fei, Ye & Li, 2001, was selected as the outgroup. Each of the 13 PCGs was extracted from the dataset of 23 mitogenomes and checked manually. Subsequently, all PCGs were aligned using the inbuilt MUSCLE module in MEGA and then concatenated to create a combined PCGs dataset. The 13 PCGs concatenated dataset was used to reconstruct the phylogenetic tree using Bayesian inference (BI) (Huelsenbeck and Ronquist 2001) and maximum likelihood (ML) (Höhler et al. 2022) methods. The optimal partition scheme and model for BI and ML were identified using PartitionFinder 2 (Lanfear et al. 2017). BI analysis was performed in MrBayes 3.2.6 with a run set to 10 million generations, sampled every 1000 generations. The initial 25% of the tree topologies were discarded as burn-in, and the consensus tree and posterior probabilities were calculated from the remaining trees. ML analysis was conducted using RAxML 8.2.0, and the support values of the tree were assessed by conducting a bootstrap test with 1000 replicates. The phylogenetic tree was visualized, checked, and improved using FigTree 1.4.2 (Rambaut 2009).

### ﻿Gene rearrangement analysis

Gene rearrangement in *Rana* was analyzed by comparing the gene orders across the entire mitogenomes of the 22 species in *Rana*, most of which were assembled using NGS techniques (Van Dijk et al. 2014; Ye et al. 2014). The two species we assembled in this study were compared in detail with each other and within the broader context of gene rearrangement patterns in the *Rana* mitogenome dataset. Based on the complete mitogenomes downloaded from GenBank, we reidentified the tRNAs of each species using tRNAscan-SE (Chan and Lowe 2019) to further verify the gene orders of tRNAs. TBtools (Chen et al. 2023) was also employed to assist in the gene rearrangement analysis.

## ﻿Results

### ﻿Mitogenome assembly and annotation

**Figure 1.**
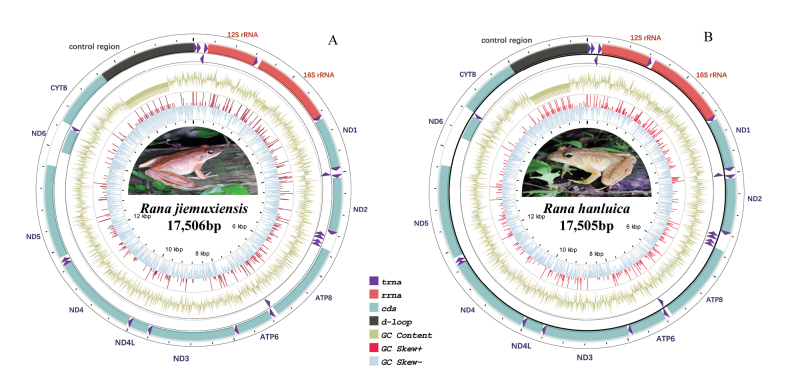
Mitodondrial gene maps of **A***R.jiemuxiensis* and **B***R.hanluica*.

The complete mitogenomes of *R.jiemuxiensis* and *R.hanluica* were circular DNA molecules with lengths of 17,506 bp and 17,505 bp, respectively (Fig. 1). Both species exhibited a typical mitogenome organization, consisting of 37 genes, including 13 PCGs, 22 tRNA genes, two rRNA genes, and one control region (CR) (Table 1). All genes were encoded by the heavy strand (H-strand), except for eight tRNA genes (tRNA^His^, tRNA^Phe^, tRNA^Pro^, tRNA^Leu^, tRNA^Val^, tRNA^Gln^, tRNA^Cys^, tRNA^Tyr^) and one PCG (ND6), which were encoded by the light strand (L-strand). The final complete mitogenomes, along with annotated information for both species, have been deposited in GenBank under accession numbers PP228843 and PP228844.

**Table 1.** Characteristics of the mitogenomes of *R.jiemuxiensis* (RJ) and *R.hanluica* (RH).

Gene	Position				Length		Strand^*^	Codons				Anti codon	Intergenic nucleotide^＃^	
	RJ		RH		RJ	RH		RJ		RH			RJ	RH
	From	To	From	To				Start codons	Stop codons	Start codons	Stop codons			
tRNA^Leu^ (CUN)	1	74	1	74	74	74	H	–	–	–	–	TAG	0	0
tRNA^Thr^	75	144	75	144	70	70	H	–	–	–	–	TGT	0	0
tRNA^Pro^	145	213	145	213	69	69	L	–	–	–	–	TGG	1	1
tRNA^Phe^	215	286	215	285	72	71	H	–	–	–	–	GAA	0	0
12S rRNA	287	1216	286	1215	930	930	H	–	–	–	–	–	0	0
tRNA^Val^	1217	1285	1216	1284	69	69	H	–	–	–	–	TAC	0	0
16S rRNA	1286	2860	1285	2859	1575	1575	H	–	–	–	–	–	0	0
tRNA^Leu^ (UUR)	2861	2934	2860	2933	74	74	H	–	–	–	–	TAA	47	47
ND1	2982	3935	2981	3934	954	954	H	ATT	AGG	ATT	AGG	–	–41	–41
tRNA^Ile^	3895	3965	3894	3964	71	71	H	–	–	–	–	GAT	1	0
tRNA^Gln^	3967	4037	3965	4035	71	71	L	–	–	–	–	TAA	–2	–2
tRNA^Met^	4036	4106	4034	4104	71	71	H	–	–	–	–	CAT	–1	–1
ND2	4106	5140	4104	5138	1035	1035	H	ATG	TAG	ATG	TAG	–	–2	–2
tRNA^Trp^	5139	5208	5137	5206	70	70	H	–	–	–	–	TCA	–1	–1
tRNA^Ala^	5208	5278	5206	5276	71	71	L	–	–	–	–	TGC	0	0
tRNA^Asn^	5279	5351	5277	5349	73	73	L	–	–	–	–	GTT	2	2
NCR	5354	5378	5352	5377	25	26	H	–	–	–	–	–	–2	–2
tRNA^Cys^	5377	5442	5376	5441	66	66	L	–	–	–	–	GCA	0	0
tRNA^Tyr^	5443	5509	5442	5508	67	67	L	–	–	–	–	GTA	1	1
COX1	5511	7064	5510	7063	1554	1554	H	GTG	AGG	GTG	AGG	–	–10	–10
tRNA^Ser^ (UCN)	7055	7126	7054	7125	72	72	L	–	–	–	–	TGA	1	1
tRNA^Asp^	7128	7196	7127	7195	69	69	H	–	–	–	–	GTC	135	0
COX2	7332	7884	7196	7883	553	688	H	ATG	T(AA)	ATG	T(AA)	–	0	0
tRNA ^(Lys|Asn)^	7885	7953	7884	7952	69	69	H	–	–	–	–	TTT	1	1
ATP8	7955	8117	7954	8115	163	162	H	ATG	TAA	ATG	TAA	–	–8	–7
ATP6	8110	8823	8109	8791	714	683	H	ATG	AGT	ATG	AGT	–	–32	–1
COX3	8792	9576	8791	9575	785	785	H	ATG	TA(A)	ATG	TA(A)	–	–2	–2
tRNA^Gly^	9575	9644	9574	9643	70	70	H	–	–	–	–	TCC	–46	–46
ND3	9599	9948	9598	9947	350	350	H	ATG	TA(A)	ATG	TA(A)	–	35	35
tRNA^Arg^	9984	10053	9983	10052	70	70	H	–	–	–	–	TCG	0	0
ND4L	10054	10338	10053	10337	285	285	H	ATG	TAA	ATG	TAA	–	–7	–7
ND4	10332	11703	10331	11702	1372	1372	H	ATG	T(AA)	ATG	T(AA)	–	–12	–12
tRNA^His^	11692	11759	11691	11758	68	68	H	–	–	–	–	GTG	0	0
tRNA^Ser^ (AGY)	11760	11826	11759	11825	67	67	H	–	–	–	–	GCT	32	31
ND5	11859	13646	11857	13644	1788	1788	H	ATG	AGG	ATG	AGG	–	624	305
ND6	14271	14765	13950	14444	495	495	L	ATG	AGA	ATG	AGA	–	0	0
tRNA^Glu^	14766	14834	14445	14513	69	69	L	–	–	–	–	TTC	3	3
CYTB	14838	15980	14517	15659	1143	1143	H	ATG	TAA	ATG	TAA	–	0	0
D-Loop	15981	17505	15660	17505	1525	1846	H	–	–	–	–	–	0	0

* H and L indicate genes transcribed on the heavy and light strand, respectively. # Positive numbers correspond to the nucleotides separating adjacent genes; negative numbers indicate overlapping nucleotides.

In *R.jiemuxiensis* and *R.hanluica*, 13 distinct but overlapping sites were found in their mitogenomes. Three of these overlapping sites were observed between contiguous PCGs, ATP8 and ATP6, ATP6 and COX3, and ND4L and ND4, respectively. Ten gene intervals were also identified, with the largest one located between ND5 and ND6 in both species, measuring 624 bp and 305 bp, respectively. The shortest interval identified was only 1 bp, found in multiple locations within both species. The lengths of the 13 PCGs varied considerably. The longest gene was ND5, which was identical in length in both species at 1788 bp, while the shortest gene was ATP8, with lengths of 162 bp and 163 bp for *R.jiemuxiensis* and *R.hanluica*, respectively. Both species had ATG as the start codon for most PCGs, except for ND1 (ATT) and COX1 (GTG). Five typical stop codons, including TAG, AGG, AGA, AGT, and TAA, as well as two kinds of incomplete terminal codons (TA-, T-), were found in the PCGs within their mitogenomes.

### ﻿Nucleotide composition and diversity

The overall base composition of *R.jiemuxiensis* was as follows: A (24.33%), T (29.11%), G (15.65%), C (30.49%), while that of *R.hanluica* was A (24.47%), T (29.37%), G (15.65%), C (30.49%). Both species showed an A+T bias with greater A+T than G+C content. Additionally, both species exhibited negative AT skew and GC skew, indicating a predominant bias towards T and C base pairs. The mitogenome sequences of the 22 *Rana* species compiled in this study ranged from approximately 16,000 bp to 22,000 bp in length, indicating a complex mitogenome evolution among *Rana* species. However, all species showed a similar A+T content bias and T and C base pair biases that resembled *R.jiemuxiensis* and *R.hanluica* (Table 2).

**Table 2.** Basal composition (percentage) of the mitogenomes of *R.jiemuxiensis* and *R.hanluica* and 20 other *Rana* species.

Name	T%	C%	A%	G%	Total length	(A+T)%	GC skew	AT skew	Accession number
*R.hanluica*	29.37567	30.49626	24.47528	15.65279	17505	53.85094	–0.32164	–0.091	PP228844*
*R.jiemuxiensis*	29.11775	30.73639	24.33298	15.81288	17506	53.45073	–0.3206	–0.08952	PP228843*
*R.dybowskii*	30.53428	29.31434	24.57703	15.57435	18864	55.11131	–0.30609	–0.1081	KF898355
*R.chensinensis*	30.69457	29.10062	24.94212	15.26269	18808	55.63669	–0.31192	–0.10339	KF898356
*R.draytonii*	29.67805	30.06937	25.37353	14.87905	17805	55.05158	–0.33795	–0.07819	KP013110
*R.huanrensis*	30.76512	29.04605	25.06458	15.12425	19253	55.8297	–0.31518	–0.10211	KT588071
*R.amurensis*	30.9651	29.11325	25.5609	14.36075	18470	56.526	–0.33934	–0.09561	KU343216
*R.chaochiaoensis*	30.19221	29.76508	24.68411	15.3586	18591	54.87631	–0.31927	–0.10037	KU246048
*R.kukunoris*	30.70901	29.11663	25.06005	15.11431	18863	55.76906	–0.31657	–0.10129	KU246049
*R.omeimontis*	29.75714	30.03292	24.16155	16.04839	19934	53.91869	–0.30347	–0.10378	KU246050
*R.temporaria*	30.66239	29.20228	24.90207	15.23326	16061	55.56446	–0.31437	–0.10367	MH536744
*R.uenoi*	30.60552	29.439	24.87979	15.07569	17370	55.48531	–0.32266	–0.10319	MW009067
*R.johnsi*	29.40915	30.72611	25.2981	14.56665	17837	54.70724	–0.35678	–0.07515	MZ571365
*R.dabieshanensis*	29.61744	30.22242	24.1637	15.99644	18291	53.78114	–0.3078	–0.10141	MW526989
*R.hanluica*	29.37461	30.5133	24.4907	15.62139	19395	53.86531	–0.32279	–0.09067	MZ680529
*R.zhenhaiensis*	29.16111	30.59336	24.66862	15.57691	18806	53.82973	–0.32524	–0.08346	OL681880
*R.wuyiensis*	29.43584	30.66382	25.44937	14.45097	17779	54.88521	–0.35937	–0.07263	OL467321
*R.catesbeiana*	32.8264	26.68979	25.99609	14.48773	17212	58.82248	–0.29633	–0.11612	ON746668
*R.arvalis*	30.69122	29.13442	24.81986	15.35451	16143	55.51108	–0.30974	–0.10577	MT872666
*R.coreana*	29.22448	30.46958	24.7243	15.58164	22262	53.94877	–0.32329	–0.08342	ON920705
*R.longicrus*	31.33066	28.63373	25.73209	14.30352	17833	57.06275	–0.33375	–0.09811	MZ680528
*R.kunyuensis*	31.27726	28.63373	25.8834	14.20561	22255	57.16066	–0.3368	–0.09436	KF840516
Avg.	30.24552	29.62343	24.96542	15.16564	18493	55.21094	–0.3228	–0.09564	–

*, the sequence obtained in this study.

When examining the average length and nucleotide composition of each PCG (Table 3), there were generally similarities, but differences remained. The shortest PCG was ATP8, and the longest one was ND5. The ND6 gene exhibited distinct differences in AT skew and GC skew compared to other PCGs. After removing the stop codon from each PCG, the total aligned length of the final 13 PCGs dataset was 11,244 bp. Nucleotide diversity analysis of each PCG showed values ranging from 0.18 (COX3) to 0.27 (ND5). In addition to ND5 (0.27), four other PCGs exhibited relatively high nucleotide diversity, namely ND3 (0.24), ATP6 (0.24), ND2 (0.25), and ATP8 (0.26), while the remaining PCGs showed relatively low nucleotide diversity, all less than 0.2.

**Table 3.** The average proportion of 13 PCGs in 22 *Rana* species.

Gene	T%	C%	A%	G%	(A+T)%	Total length	GC skew	AT skew
ND1	31.8	31.5	23.7	13	55.5	958	–0.41964	–0.14595
ND2	29	32	27.7	11.3	56.7	1029	–0.43921	–0.02293
COX1	29.7	28	24.8	17.4	54.5	1684	–0.26115	–0.08991
COX2	26.3	27.5	29.7	16.5	56	548	–0.22897	0.060714
ATP8	28.9	28.8	32.1	10.1	61	159	–0.48205	0.052459
ATP6	31.5	31.9	25.4	11.2	56.9	682	–0.47541	–0.10721
COX3	30.1	30.4	22.7	16.8	52.8	783	–0.28358	–0.14015
ND3	33.2	31.3	21	14.6	54.2	337	–0.38912	–0.22509
ND4L	30.1	31.8	24.6	13.5	54.7	282	–0.38073	–0.10055
ND4	30.3	31	25.9	12.9	56.2	1363	–0.40278	–0.07829
ND5	30.2	29.8	25.7	14.3	55.9	1779	–0.3573	–0.0805
ND6	34.8	10.7	17.8	36.6	52.6	491	0.02521	–0.32319
CYTB	29.2	32.8	24.1	13.9	53.3	1140	–0.35499	–0.09568

### ﻿Analysis of codon usage and genetic distance

**Figure 2.**
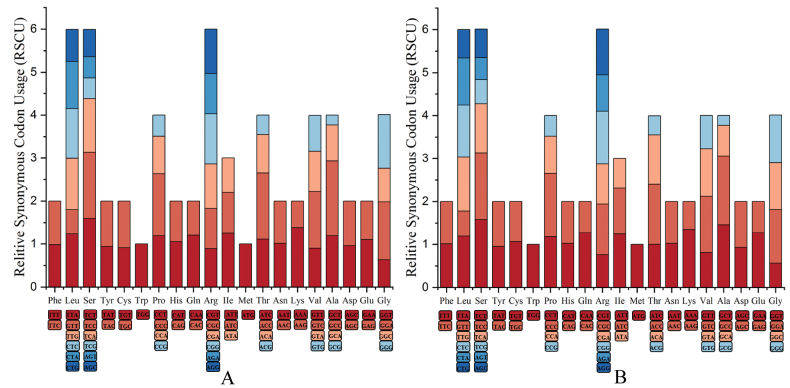
Relative synonymous codon usage in the protein coding genes of **A***R.jiemuxiensis* and **B***R.hanluica*.

There are a total of 20 amino acids encoded by the PCGs of *R.jiemuxiensis* and *R.hanluica*. Among these amino acids, Leu, Ser, and Arg had the highest frequency, while Trp and Met had the lowest. According to the RSCU analysis, Leu, Ser, and Arg were encoded by six codons each; Pro, Thr, Val, Ala, and Gly were encoded by four codons each; Phe, Tyr, Cys, His, Gln, Asn, Lys, Asp, and Glu were encoded by two codons each; Trp and Met were encoded by only one codon each. This reflects a significant bias in codon usage in their mitogenomes (Fig. 2).

**Figure 3.**
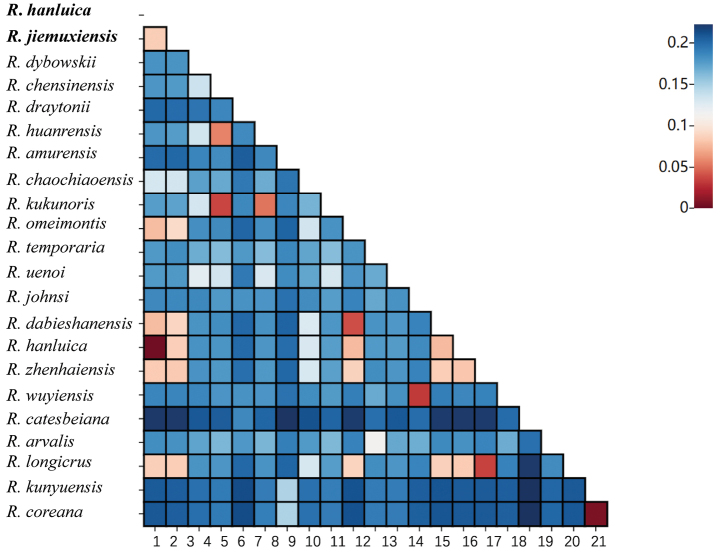
Genetic distance heatmap plot within 22 *Rana* species.

**Figure 4.**
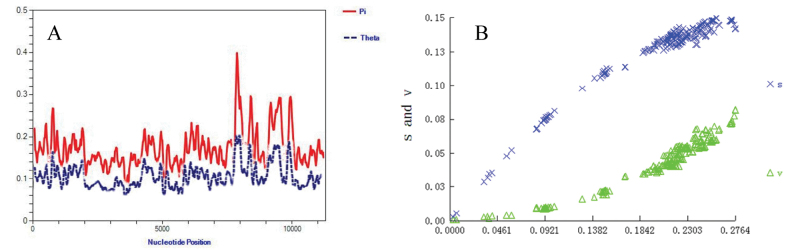
**A** Nucleotide diversities and **B** substitution saturation plot of the mitochondrial protein coding genes.

Genetic distances were analyzed among 22 *Rana* species (Fig. 3). *Ranajiemuxiensis* was found to be closely related to *R.zhenhaiensis* Ye, Fei & Matsui, 1995, *R.longicrus*, *R.hanluica*, *R.dabieshanensis* Wang, Qian, Zhang, Guo, Pan, Wu, Wang & Zhang, 2017, and *R.omeimontis* Ye & Fei, 1993, with genetic distances of 0.08327, 0.08529, 0.08547, 0.08932, and 0.09243, respectively. Conversely, *R.hanluica* was closely related to *R.dabieshanensis*, *R.omeimontis*, *R.jiemuxiensis*, *R.zhenhaiensis*, and *R.longicrus*, with genetic distances of 0.07802, 0.07793, 0.08357, 0.08398, and 0.08609, respectively. *Ranacatesbeiana* Dubois, 1992 appeared to be the most genetically distant from all other *Rana* species, suggesting it may be an ancient ancestor species. Sliding window analysis along the concatenated PCGs dataset showed significant variation in nucleotide diversity (Pi) among different genes (Fig. 4A). Substitution saturation test indicated that *Iss* < *Iss. c* in general, and scatter diagrams also suggested that the PCGs substitution was not saturated, making them suitable for phylogenetic analysis (Fig. 4B).

**Figure 5.**
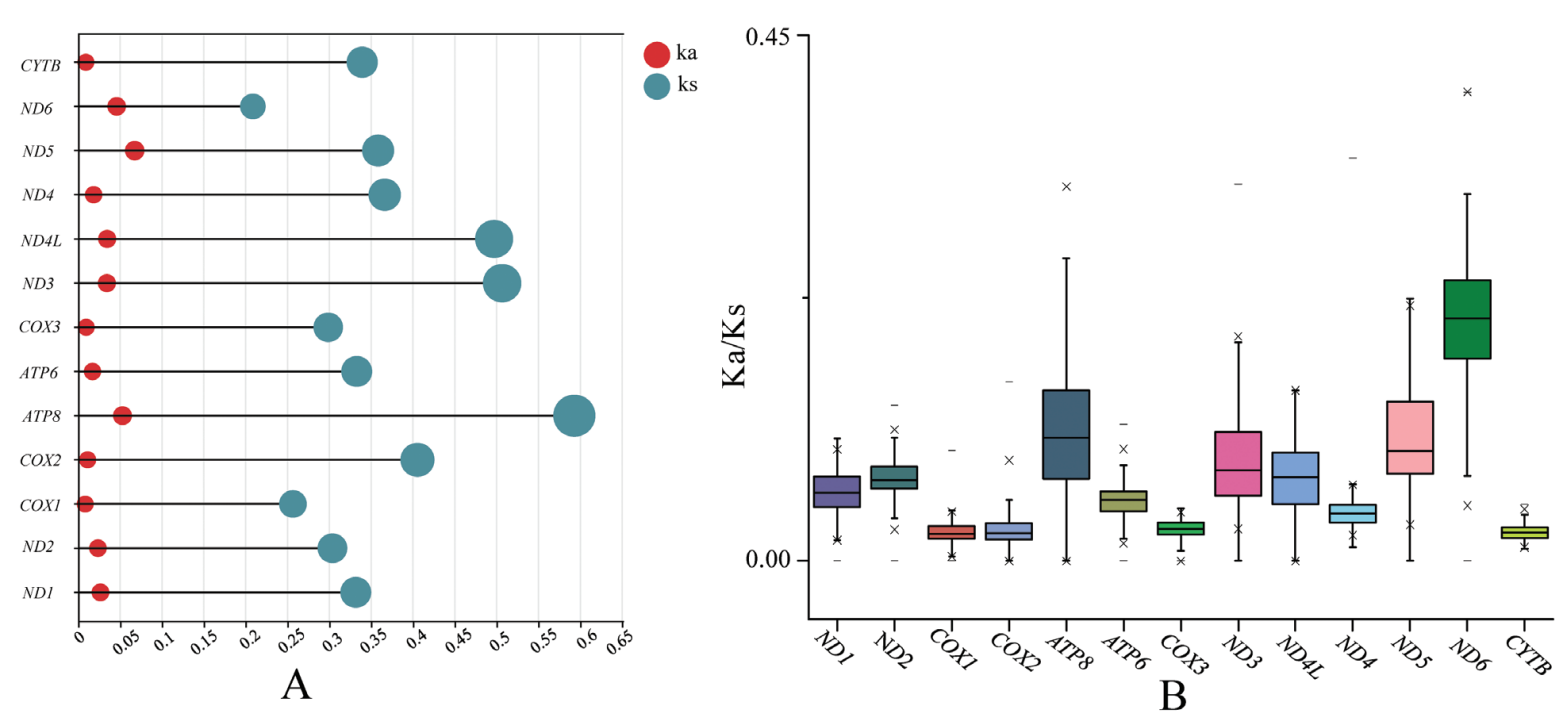
Standard deviation of **A** Ks and Ka and **B** the Ka/Ks ratio of 13 protein coding genes.

Standard deviations of Ka and Ks for the 13 PCGs across 22 species showed that the data were generally concentrated, with variances of Ks generally greater than those of Ka (Fig. 5A). ATP8 and ND6 exhibited the highest and lowest standard deviations of Ks, respectively, while ND5 and COX1 showed the highest and lowest standard deviations of Ka, respectively. The average Ka/Ks values from pairs of species among the 22 *Rana* species fell into the range of 0 to 1. Among the 13 PCGs analyzed, ND6 and ATP8 genes evolved relatively quickly, exhibiting the highest Ka/Ks values. Conversely, COX1 and CYTB had the lowest Ka/Ks ratios (Fig. 5B). However, all 13 PCGs had Ka/Ks values below 0.45, with no signals of positive selection detected.

### ﻿Phylogenetic analysis

**Figure 6.**
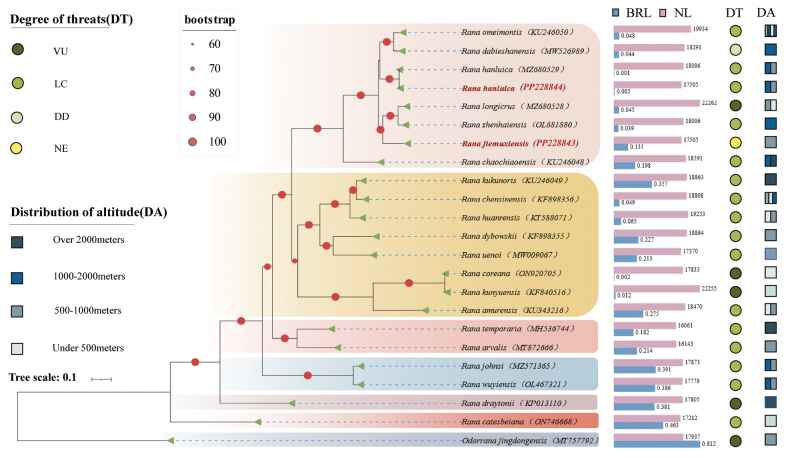
Phylogenetic tree of *Rana* species based on BI and ML analyses. Note: samples sequenced in this study are highlighted in red. BRL represents the length of the evolutionary branch and NL represents the length of the nucleotide sequence.

The tree topologies resulting from BI and ML analyses were identical, with only slight differences in the support values of some nodes (Fig. 6). Generally, the support values were high in most branches. Despite their sympatric coexistence, *R.jiemuxiensis* and *R.hanluica* did not cluster together in the same subclade. *Ranajiemuxiensis* was grouped with *R.longicrus* and *R.zhenhaiensis* to form one subclade, while *R.hanluica* was grouped with *R.omeimontis* and *R.dabieshanensis* in another subclade. However, the subclades containing *R.jiemuxiensis* and *R.hanluica* were then grouped together to form a major clade. The phylogenetic tree also revealed five other major clades, with *R.catesbeiana* and *R.draytonii* Baird & Girard, 1852 representing the two most ancient clades. Evolutionary branch lengths reflected the evolutionary history of each branch, indicating that earlier clades had longer branch lengths. Sequence lengths, distribution of altitude, and degree of threatened levels, however, did not show strong links to the phylogenetic relationships, indicating a complicated and specific evolutionary history for each species.

### ﻿Gene rearrangement analysis

**Figure 7.**
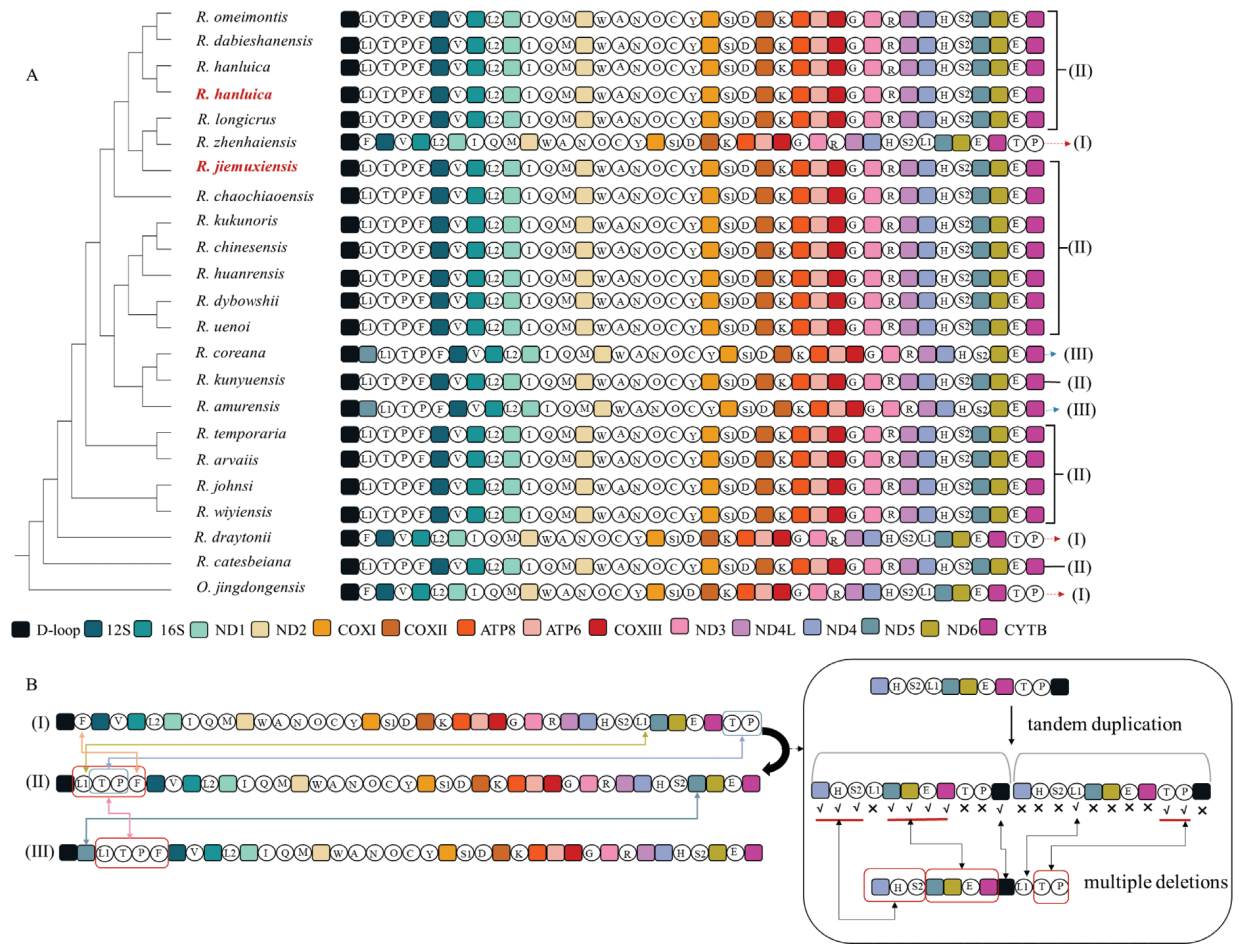
**A** Phylogenetic gene orders within 22 *Rana* species and **B** three patterns of mitochondrial gene rearrangement. Note: The icons represent tRNAs as: (1) L1: tRNA^Leu^ (CUN), (2) T: tRNA^Thr^, (3) P: tRNA^Pro^, (4) F: tRNA^Phe^, (5) V: tRNA^Val^, (6) L2: tRNA^Leu^ (UUR), (7) I: tRNA^Ile^, (8) Q: tRNA^Gln^, (9) M: tRNA^Met^, (10) W: tRNA^Trp^, (11) A: tRNA^Ala^, (12) N: tRNA^Asn^, (13) O: NCR, (14) C: tRNA^Cys^, (15) Y: tRNA^Tyr^, (16) S1: tRNA^Ser^ (UCN), (17) D: tRNA^Asp^, (18) K: tRNA^(Lys|Asn)^, (19) G: tRNA^Gly^, (20) R: tRNA^Arg^, (21) H: tRNA^His^, (22) S2: tRNA^Ser^ (AGY), (23) E: tRNA^Glu^.

As expected, the mitogenomes of *Rana* species exhibited substantial gene rearrangements and were categorized into three distinct patterns (Fig. 7A). Only two species, *R.draytonii* and *R.zhenhaiensis*, retained the first pattern, which is assumed to be ancient based on its presence in most amphibian species, including the relatively close outgroup species *Odorranajingdongensis* used in this study, as well as species from other studies such as *Leptobrachium* and *Boulenophrys* species in Anura (Zhou et al. 2023; Xiang et al. 2024) and *Tylototriton* species in Caudata (Wang et al. 2022). Interestingly, 18 out of the 22 examined *Rana* species shared the second and also the most dominant pattern, accounting for 82% of the species analyzed. Both *R.jiemuxiensis* and *R.hanluica*, sequenced in this study, belonged to this dominant pattern. Two other species, *R.amurensis* Boulenger, 1886 and *R.coreana* Okada, 1928, shared the third, rare pattern, which is similar to the second pattern except that the ND5 gene is transposed to a position behind the control region (D-loop). The transition from the first pattern to the second and third patterns primarily resulted from changes in the positions of tRNA genes. The gene orders of rRNAs remained unchanged, except for the rearrangement of ND5 from the second to the third pattern.

We proposed a plausible scenario to explain the mitochondrial gene rearrangements within *Rana*, considering that duplications and losses are more likely to occur among tRNAs than rRNAs and PCGs. According to the principle of parsimony, a “tandem duplication - multiple deletion” event in a sequence region spanning from ND4 to the D-loop likely triggered the transition from the first ancient pattern to the second pattern. Subsequently, a simple transposition of ND5 would have driven the second pattern to evolve into the third pattern (Fig. 7B). In the evolutionary history of *Rana*, for reasons yet unclear, the second pattern became the dominant pattern within this group. This dominance pattern in mitochondrial gene orders in *Rana* is distinct from those in other amphibian species.

### ﻿Species verification based on individual genes

Species verification is crucial for publishing the complete mitogenome of a species. To verify the molecular identification of the species involved in this study, we selected the individual 16S rRNA, ND2, and CYTB genes as target genes because they have abundant resources in NCBI based on previous studies (Djebbi et al. 2018). These genes are also frequently used in DNA barcoding studies (Formenti et al. 2021; Ahmed et al. 2022). Through BLAST homology searching operations, the CYTB, ND2, and 16S rRNA fragments from our samples showed high similarities, 100%, 99.64%, and 99.79%, respectively, with the species named *R.jiemuxiensis* in NCBI, which was collected from its type locality in Yuanling County, Hunan Province. Similarly, the gene fragments of *R.hanluica* samples showed nearly 100% similarity with these genes of *R.hanluica* collected from Lishui City, Zhejiang Province. It is generally accepted that gene similarities between individuals of the same species are usually greater than 98%. Therefore, we conclude that the two species used in this study were correctly identified.

## ﻿Discussion

### ﻿Characteristics of the mitogenomes

Mitochondrial DNAs, or mitogenomes, often serve as valuable molecular markers and have been widely applied in molecular biology and ecological studies. Typically, animal mitogenomes contain 2 rRNAs (12S and 16S rRNA), 22 tRNAs, 13 PCGs, and a control region (also known as the D-loop), with a sequence length usually ranging from 16 to 17 kb (Huang et al. 2016; Tang et al. 2021). Vertebrate mitogenomes are particularly conservative with several unique characteristics, including maternal inheritance, a rapid evolutionary rate, and low levels of recombination. These characteristics make mitochondrial DNA valuable in reconstructing phylogenetic relationships, testing selective pressures, and identifying species using mitochondrial barcoding genes, etc. (Jiang et al. 2023; Lan et al. 2023; Zhang et al. 2023; Xiang et al. 2024).

The utilization of mitochondrial DNA in molecular identification provides a valuable tool in taxonomic studies compared to traditional morphological approaches (Li et al. 2021). In recent years, an increasing number of species identifications have relied on the combination of morphology and molecular evidence. In some cases, such as cryptic species identifications, molecular evidence carries more weight than morphological evidence. Molecular data are commonly used in species identification and phylogenetic studies (Miya and Nishida 2015; Wang et al. 2016; Tan et al. 2018), especially with the rapid advancements in NGS technology. The two sympatric *Rana* species involved in this study, *R.jiemuxiensis* and *R.hanluica*, are morphologically similar, with a distinguishing characteristic primarily based on the number of bands on the thighs and tibia (5 or 6 in *R.jiemuxiensis* vs 8 or 9 in *R.hanluica*). In the field, these two species often seem to completely overlap, as they are sometimes found coexisting in very small areas. However, their genetic distance and phylogenetic position are distinct (Fig. 3 and Fig. 6, respectively), which is also evident in the species verification based on BLAST searches of our sequences against the NCBI database.

The nucleotide composition of both *R.jiemuxiensis* and *R.hanluica* exhibited a distinct A+T rich pattern (Table 3), which is commonly observed in the mitogenomes of other species (Rayko 1997; Françoso et al. 2023). This typical nucleotide composition is highly conserved among vertebrates (Zhou et al. 2006; Hao et al. 2016; Vinogradov and Anatskaya 2017). In *Rana* species, the A+T content generally exceeds 50% but is less than 60%. The gene structure of both sequenced species was identical and similar to other animal species, with the majority of genes, especially the PCGs, located on the H strand, while only a few are on the L strand (Boore 1999). There were 13 gene overlaps in the mitogenomes of *R.jiemuxiensis* and *R.hanluica*, which is also common in other *Rana* species (Zyla et al. 2019), indicating the compact nature of mitogenomes in regulating gene expression. However, gene intervals were also commonly present in frog mitogenomes, with the locations varying more than overlapping regions and showing species-specific patterns. For example, for the two *Rana* species here, the gene interval between tRNA^Leu^ and ND1 was 41 bp, whereas there was no interval between 12S rRNA and tRNA^Val^. In contrast, in the mitogenomes of two *Leptobrachium* species (Zhou et al. 2023), there was no interval between tRNA^Leu^ and ND1 genes, and the interval between 12S rRNA and tRNA^Val^ was 4 bp. The usage of start and stop codons in different mitogenomes also showed species-specific patterns, which have received limited attention (Wang et al. 2022). In the two *Rana* mitogenomes sequenced here, all genes shared the start codon ATG, except for COX1 (GTG) and ND1 (ATT). In comparison, the start codons of COX1, ATP6, and ATP8 were GTG in the mitogenomes of two *Tylototriton* species. Three rare types of stop codons (AGT, AGA, and AGG) were also identified in this study, which are less common but have been observed in other *Rana* species.

The ratio of Ka and Ks is a popular proxy for detecting adaptive evolution, with Ka/Ks > 1 reported in the mitochondrial PCGs of some species (Ye et al. 2017; Bi et al. 2020). However, none of the Ka/Ks values from the PCGs within *Rana* species exceeded 1 (Fig. 5A), indicating that the overall evolution pattern of *Rana* mitogenomes tends to be conservative in maintaining the functions of regularly generated proteins. The relatively high Ka/Ks ratios observed in some PCGs may represent higher evolutionary rates, such as those of the ATP6, ND3, ND5, and ND6 genes (Fig. 5B). These mitochondrial PCGs that evolve more rapidly may accumulate advantageous mutations, potentially enhancing the fitness of the species in adapting to environmental changes (Yang et al. 2018).

### ﻿Phylogenetic implications and gene rearrangement evolution

Mitochondrial DNA sequences, particularly mitogenomes, are increasingly utilized in phylogenetic studies (Dhorne-Pollet et al. 2020). In this study, a phylogenetic tree of the genus *Rana* was constructed based on 13 mitochondrial PCGs from 22 species, representing the most comprehensive and detailed mitogenomic phylogenetic tree of *Rana* to date. Although coexisting in Zhangjiajie National Forest Park, *R.jiemuxiensis* and *R.hanluica* were found in distinct phylogenetic lineages (Fig. 6). *Ranajiemuxiensis* clustered with *R.longicrus* and *R.zhenhaiensis* to form a subclade, while *R.hanluica* grouped with *R.omeimontis* and *R.dabieshanensis* in another subclade. This supports our speculation that the two sympatric species are genetically separated to a certain extent. Despite including more species, our phylogenetic tree was largely consistent with previous studies that also utilized mitogenomes in *Rana* (Chen et al. 2018; Wang et al. 2020; Zhang et al. 2020).

The differentiation of species in *Rana* is possibly associated with geographic isolation and habitat selection. The known distribution altitudes of *Rana* species were mapped onto the phylogenetic tree (Fig. 6). It revealed that *Rana* species have a wide altitudinal adaptation, ranging from over 100 meters to more than 2000 meters above sea level. However, no clear correlations were evident between phylogenetic position and distribution altitude ranges. Previous studies have shown that the skin morphological properties of *Rana* species at high altitudes differ from those at low altitudes (Zhi and Li 2016). It was further inferred that amphibians living at high altitudes, thriving in low temperatures, may have slower evaporative rates (Zyla et al. 2019). Additionally, the degree of threat for each *Rana* species, acquired from the IUCN Red List of Threatened Species of Amphibians, was also mapped onto the phylogenetic tree. Among the 22 *Rana* species in this study, five were classified as vulnerable (VN), 15 as least concern (LC), three as data deficient (DD), and four as not evaluated (NE). Generally, the species in *Rana* are experiencing a medium level of threats.

Previous studies have revealed that interspecies variations in mitochondrial gene orders are prone to occur in certain groups (Shao and Barker 2003; Liu et al. 2017), including species within *Rana* (Zhu et al. 2018). However, few studies have analyzed mitogenomic gene rearrangements in *Rana* from a phylogenetic perspective. By examining the gene orders of 22 *Rana* species in this study, three distinct patterns can be discerned (Fig. 7A). The first pattern represents a very ancient type that is shared with most amphibian species (Wang et al. 2022; Zhou et al. 2023). The second pattern, however, is dominant in *Rana* species and mainly results from position changes of some tRNAs in their mitogenomes. The third pattern appears to be derived from the second one, with only one PCG (ND5) seemingly transposed. Interestingly, the three patterns of gene arrangements show a trend of parallel evolution, with none of them being confined to a single subclade.

A simple parsimony scenario is proposed to explain the evolutionary process of the three patterns observed in this study (Fig. 7B), which we refer to as the “tandem duplication-multiple deletion” hypothesis. This phenomenon is commonly observed in mitogenomes, where tandem duplications can occur due to strand slippage during replication, followed by gene deletions under selective pressures (Moritz and Brown 1987; Levinson and Gutman 1987). In the case of *Rana* mitochondrial genes, this duplication and loss event can drive the evolution from the first ancient pattern to the second dominant pattern. The genes involved in this process include 6 tRNAs, 4 PCGs, and a control region, with changes mainly resulting from the tRNAs. Previous studies have indicated that tRNAs in mitogenomes are relatively neutral (Dowton et al. 2009; Lavrov and Pett 2016), suggesting that gene arrangements resulting from tRNA position changes may not significantly alter the conservative functions of mitochondria. Additionally, tandemly duplicated regions often favor genes that utilize stem-loop structures during replication (Stanton et al. 1994), a characteristic typical of vertebrate tRNAs (Macey et al. 1997). The involvement of one PCG (ND5) from the second to the third pattern echoes that changes in tRNAs and PCG positions are frequently observed in mitochondrial gene rearrangements in both invertebrates and vertebrates (Cantatore et al. 1989; Miya and Nishida 1999).

The study of gene rearrangement patterns can provide insights into taxonomy and elucidate the complex evolutionary history among species (Ye et al. 2021; Feng et al. 2023). However, a comprehensive phylogenetic tree comprising most species is necessary to fully understand the evolutionary background that underlies the gene rearrangements in *Rana*. As more mitogenomes, such as those of the two species in this study, become available in the future, we anticipate that the complete evolutionary history of *Rana* will gradually be unveiled.

## ﻿Additional information

### Author ORCIDs

Hongmei Xiang ￼ https://orcid.org/0009-0006-3251-3726

Wansheng Jiang ￼ https://orcid.org/0000-0002-6498-944X

### Data availability

The final complete mitogenomes, along with annotated information for both species, have been deposited in GenBank under accession numbers PP228843 and PP228844. All the analyses and findings of this study were based on these two sequences and other sequences that were available in GenBank.
